# Performance of continuous glucose monitoring-based meal detection algorithms in young healthy adults

**DOI:** 10.1038/s41598-026-50699-5

**Published:** 2026-05-19

**Authors:** Christoph Höchsmann, Jonas T. Weber, Sieglinde Hechenbichler Figueroa, Elizabete Laivina, Karsten Koehler

**Affiliations:** Department of Health and Sport Sciences, TUM School of Medicine and Health, Technical University of Munich, 80809 Munich, Germany

**Keywords:** Digital dietary assessment, Eating behavior monitoring, Automated nutrition tracking, Algorithm validation, Continuous glucose monitoring (CGM), Health care, Medical research

## Abstract

Continuous glucose monitoring (CGM) enables automated detection of eating events via meal detection algorithms (MDAs); however, CGM-only MDAs have not been comprehensively evaluated using a shared dataset. We compared nine published CGM-only MDAs using standardized metrics by testing them with CGM data from 16 young, healthy, normal-weight adults under free-living conditions. We employed a per-participant holdout design, with separate training, validation, and testing sets, and assessed performance on the test set (216 meals) using sensitivity, false positives per day (FP/day), and detection time (Δt). Sensitivity ranged from 49 to 90%, FP/day from 0.12 to 2.42, and Δt from 37 to 61 min. Fuzzy logic and simulation-based approaches showed the highest sensitivity (90% and 83%) but slower detection (> 59 min) and higher FP rates (> 1.28/day). Pattern-recognition classifiers (82%, 0.39 FP/day, 44 min; 77%, 0.33, 42 min) and a glucose-insulin-model-based method (77%, 0.22, 41 min) showed more balanced performance, while rate-of-change detectors were faster (37–38 min) but less sensitive (70–72%). No single MDA consistently outperformed others across metrics. Pattern-recognition and physiological modeling approaches demonstrated the most balanced performance, whereas rate-of-change methods enabled faster detection with reduced accuracy. Algorithm choice should reflect application priorities, such as early detection versus reliability.

**Supplementary Information:**

The online version contains supplementary material available at10.1038/s41598-026-50699-5.

## Introduction

Accurate assessment of eating behavior is crucial for understanding dietary patterns and energy balance; however, traditional self-report methods are limited by recall bias, underreporting, and participant burden^1^. Continuous glucose monitoring (CGM) provides an objective means of detecting eating events by capturing postprandial glucose dynamics at 1- to 5-min intervals^2^. While originally developed for diabetes management and artificial pancreas systems^3–9^, CGM is increasingly used in research and among healthy individuals to monitor eating behavior and support digital nutrition applications^10,11^.

Multiple meal detection algorithms (MDAs) have been proposed to detect meal onsets from CGM data^3,11–17^. When restricted to CGM-only input, these include rate-of-change (ROC) detectors^3,15^, fuzzy logic^18^, observer-based models^16^, supervised machine learning^11,13^, and physiology-based glucose–insulin modeling^12,14^. However, published MDAs have typically been evaluated in isolation, often using different datasets, study populations, and evaluation criteria, which limits their comparability.

Standardized metrics such as sensitivity, false positives per day (FP/day), and detection time (Δt) have been recommended for performance assessment^2^. Reported sensitivity values > 90% and < 1.5 FP/day have been achieved by several MDAs^7,12–16,18^, while detection times often exceed 30 min^3,7,13,14,16,18^. Nevertheless, few studies have applied these metrics to systematically compare multiple CGM-only MDAs under free-living conditions using a shared dataset, limiting insight into relative performance trade-offs^7,13,19^.

To address this gap, we systematically implemented and validated nine published CGM-only MDAs using CGM data from young, healthy adults in free-living conditions. We focused on CGM-only approaches because our primary interest was behavioral meal detection for nutrition monitoring and digital interventions, where methods based on a single input stream may offer greater practicality and scalability than multimodal approaches. This focus is also relevant in healthy, normoglycemic individuals, in whom smaller postprandial glucose excursions make meal detection more challenging. We evaluated performance using standardized metrics within a participant-level holdout design. By benchmarking diverse detection approaches on the same dataset and under identical evaluation conditions, we aimed to provide a reproducible comparison of algorithm performance and practical guidance for selecting appropriate MDAs depending on application priorities. Based on prior findings, we expected model-based and pattern-recognition classifiers to balance sensitivity and detection latency more effectively than ROC-based methods.

## Results

### Hyperparameter tuning

Across validation sets, F2-scores ranged from 0.60 to 0.86 (Table 1). The highest scores were observed for MDA_Dassau-2of3_ and MDA_Samadi_ (both 0.86), whereas MDA_Dassau-3of4_ showed the lowest score (0.60). Most algorithms (MDA_Dassau-2of3_, MDA_Dassau-3of4_, MDA_Faccioli_, MDA_Harvey_, MDA_Samadi_, and MDA_Turksoy_) were tuned on a per-participant basis, while MDA_Kölle-*Ra*_, MDA_Kölle-CGM_, and MDA_Popp_ were tuned using global hyperparameters. All final hyperparameter configurations derived from the validation phase were fixed prior to performance testing.

**Table 1:** Tuned hyperparameters and best F2 scores.

Meal detection algorithm	F2-score	Hyperparameter
	Mean ± SD	
MDA_Dassau−2of3_^3^	0.86 ± 0.09	Threshold_Glucose_ = 103.8 ± 5.0 Threshold_maxROC_ = 2.69 ± 0.7 Threshold_ROC_ = 1.18 ± 0.24
MDA_Dassau−3of4_^3^	0.60 ±0.16	Threshold_Glucose_ = 100.6 ± 2.5 Threshold_maxROC_ = 3.94 ± 0.77 Threshold_ROC_ = 1.01 ± 0.03 Threshold_Acceleration_ = 0.21 ± 0.03
MDA_Faccioli_^16^	0.82 ±0.09	*Th*_*Der*_ = 0.23 ± 0.11 *Th*_*Res*_ = 1.25 ± 0.54
MDA_Harvey_^15^	0.84 ±0.13	G_min_ = 103.1 ± 5.1 G’_min,2_ = 1.1 ± 0.1 G’_min,3_ = 0.8 ± 0.1 *τF* = 4.5 ± 1.2
MDA_Kölle-*Ra*_^13^	0.82 ±N/A^a^	*δ* = 10^−15^ *γ* = 0.2 *θ* = 0.70
Kölle_− CGM_^13^	0.84 ±N/A^a^	*δ* = 10^−15^ *γ* = 0 *θ* = 0.65
MDA_Popp_^12^	0.81 ±N/A^a^	*ε* = 18 *τ* = 20 *ϕ* = 0.10
MDA_Samadi_^18^	0.86 ±0.04	*γ*_1_ = 1 ± 1 Threshold_act_ = 1.33 ± 0.49
MDA_Turksoy_^14^	0.84 ±0.12	Threshold_Rα_ = 1.56 ± 0.09

^a^ Not available as these meal detection algorithms were tuned globally. Therefore, there is only one F2-score and set of parameters available for these meal detection algorithms.

Threshold_Glucose_, G_min_: Minimum glucose concentration required for a meal detection; Threshold_maxROC_: Upper threshold for the maximum rate of change of glucose; Threshold_ROC_: Threshold for the glucose rate of change; Threshold_Acceleration_: Threshold for the acceleration (second derivative) of glucose; *Th*_*Der*_: Threshold for the estimated glucose derivative; *Th*_*Res*_: Threshold for the observer-based residual signal indicating unexpected glucose rises; G’_min,2_ / G’_min,3_: Minimum required glucose rate of change over the last 2 or 3 consecutive samples; *τF*: Time constant of the low-pass filter applied to the glucose signal; *δ*: Regularization parameter controlling numerical stability of the classifier; *γ*: Weighting parameter influencing feature in the classifier; *θ*: Posterior probability threshold for classifying a time point as a meal; *ε*: Maximum allowed fit error for confirming a meal; *τ*: Maximum backward search window to determine meal start time; *ϕ*: Threshold for divergence between observed and predicted glucose trajectories; *γ*1: Gain parameter controlling sensitivity of the glucose slope in fuzzy logic classification; Threshold_act_: Activation threshold for the increase-of-glucose trend score; Threshold_Rα_: Threshold for the estimated glucose appearance rate used to detect meals.

### Performance on the test set

Table 2 summarizes algorithm-level performance across 216 meals in the test set. Sensitivity was highest for MDA_Samadi_ (89.8%), followed by MDA_Popp_ (82.9%), and MDA_Kölle-CGM_ (82.4%). Intermediate performance was observed for MDA_Kölle-*Ra*_ (77.3%), MDA_Turksoy_ (76.9%), MDA_Dassau-2of3_ (72.2%), MDA_Harvey_ (70.4%), and MDA_Faccioli_ (64.4%), whereas MDA_Dassau-3of4_ showed the lowest sensitivity (49.1%).

**Table 2:** Performance metrics of meal detection algorithms.

Algorithm	TP	FP	FN	Sensitivity (%)	FP/day	Δt (min)
MDA_Dassau−2of3_^3^	156	43	60	72.2 [65.7–78.1]	0.60 [0.43–0.80]	37.6 [33.7–41.6]
MDA_Dassau−3of4_^3^	106	9	110	49.1 [42.2–55.9]	0.12 [0.06–0.24]	36.8 [32.9–40.7]
MDA_Faccioli_^16^	139	100	77	64.4 [57.6–70.7]	1.39 [1.13–1.69]	39.6 [34.5–44.8]
MDA_Harvey_^15^	152	19	64	70.4 [63.8–76.4]	0.26 [0.16–0.41]	37.3 [34.4–40.2]
MDA_Kölle-*Ra*_^13^	167	24	49	77.3 [71.1–82.7]	0.33 [0.21–0.50]	41.7 [38.2–45.1]
MDA_Kölle-CGM_^13^	178	28	38	82.4 [76.7–87.2]	0.38 [0.26–0.56]	43.8 [40.4–47.2]
MDA_Popp_^12^	179	92	37	82.9 [77.2–87.6]	1.28 [1.03–1.57]	60.5 [55.9–65.0]
MDA_Samadi_^18^	194	174	22	89.8 [85.0–93.5]	2.42 [2.07–2.80]	58.5 [53.6–63.5]
MDA_Turksoy_^14^	166	16	50	76.9 [70.6–82.3]	0.22 [0.13–0.36]	40.7 [37.9–43.5]

Data are mean [95% CI].

*MDA* meal detection algorithm, *TP* true positives, *FP* false positives, *FN* false negatives, *FP/day* false positives per day, *Δt* detection time.

FP/day were lowest for MDA_Dassau-3of4_ (0.12), MDA_Turksoy_ (0.22), and MDA_Harvey_ (0.26), moderate for MDA_Kölle-*Ra*_ (0.33), MDA_Kölle-CGM_ (0.39), and MDA_Dassau-2of3_ (0.60), and highest for MDA_Popp_ (1.28), MDA_Faccioli_ (1.39), and MDA_Samadi_ (2.42).

Δt was shortest for MDA_Dassau-3of4_ (36.8 min), MDA_Harvey_ (37.3 min), and MDA_Dassau-2of3_ (37.6 min), with intermediate times for MDA_Faccioli_ (39.6 min), MDA_Turksoy_ (40.7 min), MDA_Kölle-*Ra*_ (41.7 min), and MDA_Kölle-CGM_ (43.8 min). MDA_Samadi_ (58.5 min) and MDA_Popp_ (60.5 min) showed the longest detection delays.

**Fig. 1:**
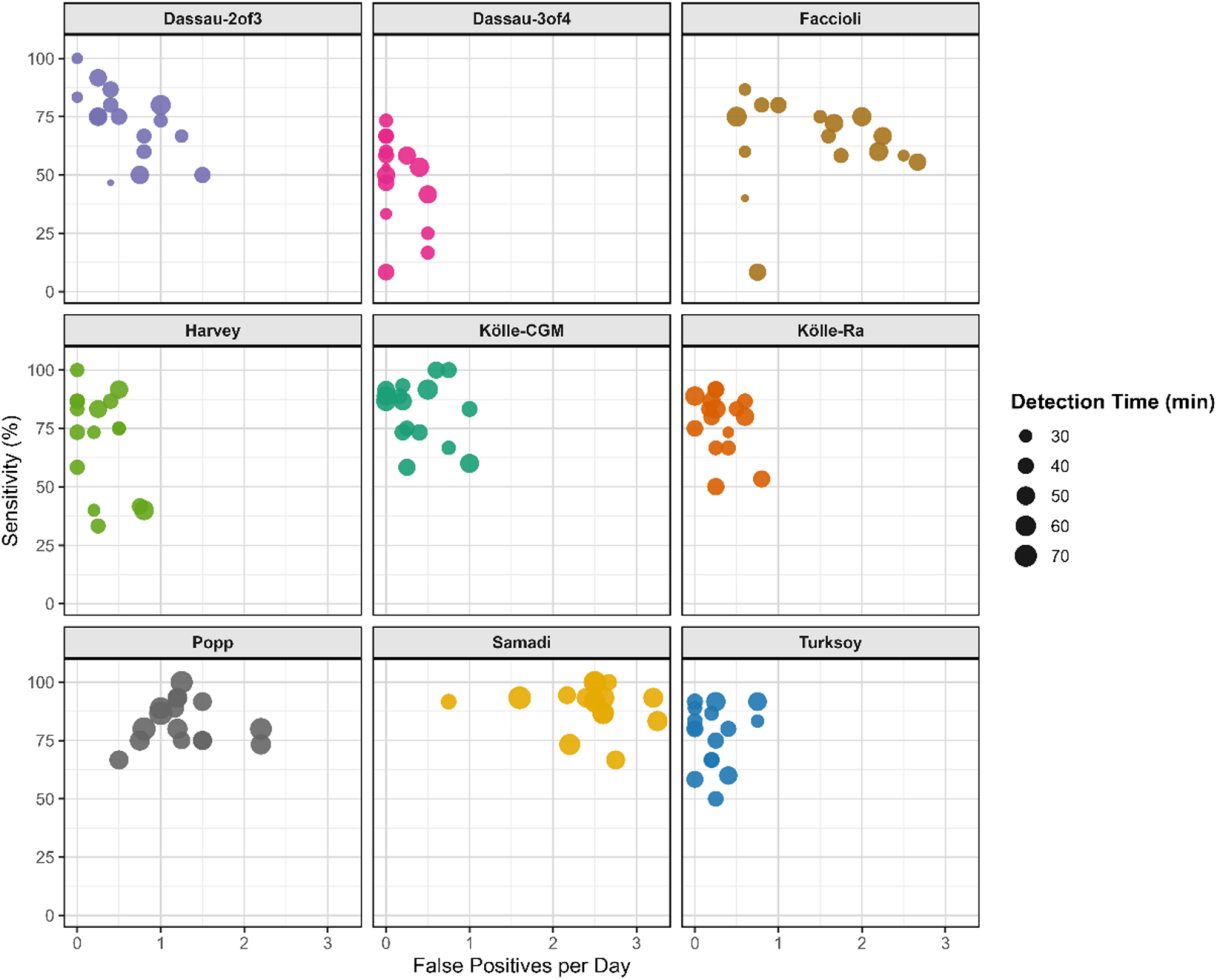
Participant-level sensitivity versus false-positives per day by meal detection algorithm. Each data point represents one participant.

Figure 1 illustrates participant-level trade-offs between sensitivity and FP/day, with point size reflecting detection time. Supplementary Table 2 shows a comparison of our observed performance values with those originally reported for each algorithm.

### Mixed-effects model results

The estimated marginal means (EMMs) with 95% CI for each algorithm are displayed in Table 3. Complete pairwise comparisons across all algorithms are provided in Supplementary Table 3.

**Table 3:** Estimated marginal means for each meal detection algorithm.

Algorithm	Sensitivity (%)	FP/day	Δt (min)
MDA_Kölle-CGM_^13^	85.2 [79.1–89.8]	0.38 [0.26–0.56]	43.8 [38.9–48.8]
MDA_Dassau−2of3_^3^	74.9 [67.2–81.3]	0.59 [0.43–0.80]	37.4 [32.3–42.4]
MDA_Dassau−3of4_^3^	48.9 [40.3–57.6]	0.12 [0.06–0.24]	37.2 [31.9–42.4]
MDA_Faccioli_^16^	66.4 [57.9–73.9]	1.37 [1.12–1.69]	39.3 [34.2–44.3]
MDA_Harvey_^15^	73.0 [65.0–79.6]	0.26 [0.17–0.41]	37.6 [32.6–42.7]
MDA_Kölle-*Ra*_^13^	80.2 [73.2–85.7]	0.33 [0.22–0.49]	41.7 [36.8–46.7]
MDA_Popp_^12^	85.7 [79.6–90.1]	1.26 [1.01–1.57]	59.1 [54.2–64.1]
MDA_Samadi_^18^	92.0 [87.4–95.0]	2.39 [2.02–2.82]	59.1 [54.1–64.0]
MDA_Turksoy_^14^	79.7 [72.7–85.3]	0.22 [0.13–0.36]	40.1 [35.1–45.1]

Data are mean [95% CI].

*MDA* meal detection algorithm, *TP* true positives, *FP* false positives, *FN* false negatives, *FP/day* false positives per day, *Δt* detection time.

#### Sensitivity model (binomial GLMM)

Fixed and random effects explained 30.7% and 20.3% of the variance, respectively. Compared with MDA_Kölle-CGM_ (85.2% [CI 79.1–89.8]), MDA_Dassau-3of4_ (48.9% [CI 40.3–57.6], OR = 0.166, *p* < 0.001), MDA_Faccioli_ (66.4% [CI 57.9–73.9], OR = 0.342, *p* < 0.001), and MDA_Harvey_ (73.0% [CI 65.0–79.6], OR = 0.468, *p* = 0.050) exhibited significantly lower sensitivity. No significant differences were observed for MDA_Dassau-2of3_ (74.9% [CI 67.2–81.3]), MDA_Kölle-*Ra*_ (80.2% [CI 73.2–85.7]), MDA_Popp_ (85.7% [CI 79.6–90.1]), MDA_Samadi_ (92.0% [CI 87.4–95.0]), or MDA_Turksoy_ (79.7% [CI 72.7–85.3]). Participants in the StandardDiet group had higher detection odds than those in the LCD group (β = 0.607, *p* = 0.020; OR = 1.84), indicating that reduced postprandial glucose excursions under carbohydrate restriction impaired meal detectability across algorithms. Weight was negatively associated with sensitivity (β = − 0.262, *p* = 0.045; OR = 0.77 per 1 SD increase; mean weight = 67.4 kg, SD = 10.0 kg). No significant effects were observed for phase, sex, height, or daily carbohydrate intake. No interactions were significant.

#### False positive model (Poisson GLMM)

Fixed and random effects explained 51.3% and 0.9% of the variance, respectively. Relative to MDA_Kölle-CGM_ (0.38 FP/day [CI 0.26–0.56]), higher FP/day were observed for MDA_Faccioli_ (1.37 [CI 1.11–1.69], IRR = 3.57, *p* < 0.001), MDA_Popp_ (1.26 [CI 1.02–1.57], IRR = 3.29, *p* < 0.001), and MDA_Samadi_ (2.39 [CI 2.02–2.82], IRR = 6.21, *p* < 0.001). Daily carbohydrate intake was positively associated with FP/day (β = 0.205, *p* = 0.005; IRR ≈ 1.23 per 1 SD [≈ 99 g] increase in carbs). No other fixed effects or interactions were significant.

#### Detection time model (LMM)

Fixed and random effects accounted for 19.8% and 17.6% of the variance. Compared with MDA_Kölle-CGM_ (43.8 min [CI 38.9–48.8]), faster detection was observed for MDA_Dassau-2of3_ (37.4 min [CI 32.4–42.4], β = − 0.324 SD, absolute difference − 6.4 min, *p* = 0.017), MDA_Dassau-3of4_ (37.2 min [CI 31.9–42.4], β = − 0.338 SD, absolute difference − 6.6 min, *p* = 0.017), and MDA_Harvey_ (37.6 min [CI 32.6–42.7], β = − 0.313 SD, absolute difference − 6.2 min, *p* = 0.022). Conversely, MDA_Popp_ (59.1 min [CI 54.2–64.1], β = + 0.771 SD, absolute difference + 15.3 min, *p* < 0.001) and MDA_Samadi_ (59.1 min [CI 54.1–64.0], β = + 0.767 SD, absolute difference + 15.3 min, *p* < 0.001) were significantly slower. No significant effects or interactions were observed for other fixed covariates.

## Discussion

This study systematically evaluated nine MDAs that rely solely on CGM signals, using free-living CGM data from young, healthy adults. The algorithms demonstrated distinct performance profiles, and no single approach performed best across all metrics, including sensitivity, FP/day, and Δt. Sensitivity ranged from 49 to 90%, FP/day from 0.12 to 2.42, and Δt from 37 to 61 min. Pattern-recognition classifiers by Kölle et al.^13^ and the glucose-insulin-model-based algorithm by Turksoy et al.^14^ provided the most balanced trade-offs, combining high sensitivity, low FP/day, and moderate Δt. In contrast, ROC-based detectors, as proposed by Dassau et al.^3^ and Harvey et al.,^15^ yielded the shortest detection times at the expense of reduced sensitivity.

Compared to their original publications, most algorithms required more permissive hyperparameters to perform well in this cohort. This likely reflects the smaller and earlier postprandial glucose excursions reported in healthy individuals compared with people with diabetes^4,20,21^. ROC-based detectors selected lower glucose thresholds and ROC criteria than originally proposed (e.g., MDA_Dassau-2of3_ selected glucose thresholds of 103.8 mg/dL vs. 150–220 mg/dL in the original work)^3^. MDA_Harvey_ similarly adopted lower ROC limits and glucose minima^15^. Glucose-insulin-model-based methods also adapted toward more permissive decision thresholds, such as a lower estimated rate of appearance threshold in MDA_Turksoy_^14^. MDA_Popp_ selected a higher error tolerance during simulation-based fitting^12^. Algorithms, such as MDA_Dassau-3of4_, which selected hyperparameters at the boundary of the predefined grid, may have performed better with an expanded tuning range.

When evaluated on the test set, MDA_Samadi_ and MDA_Popp_ achieved the highest sensitivity (92.0% and 85.7%, respectively), but at the cost of the slowest detection times (both 59.1 min) and substantially elevated FP/day (2.39 and 1.26). In contrast, MDA_Faccioli_ and MDA_Dassau-3of4_ demonstrated poor sensitivity, suggesting limited applicability in practice, particularly in clinical settings where missed detections could lead to hyperglycemia^14^. Balanced performance was seen in the pattern-recognition MDAs by Kölle et al.^13^ and the glucose-insulin-model-based MDA_Turksoy_^14^, all of which combined high sensitivity (> 79%), low FP/day (< 0.40), and moderate Δt (41–44 min). ROC-based approaches (MDA_Harvey_ and MDA_Dassau-2of3_) achieved the fastest detection times (37–38 min) with moderate sensitivity (~ 73–75%) and relatively low FP/day (< 0.60).

According to thresholds proposed by Brummer et al.^2^ (≥ 90% sensitivity and < 1 FP/day as excellent), MDA_Samadi_, MDA_Popp_, and MDA_Kölle-CGM_ met or approached the sensitivity benchmark, while MDA_Kölle-CGM_, MDA_Kölle-*Ra*_, MDA_Turksoy_, MDA_Dassau-2of3_, MDA_Dassau-3of4_, and MDA_Harvey_ met the FP/day criterion. However, all algorithms exhibited detection delays of 37 min or more, which remain suboptimal for real-time clinical decision support (e.g., artificial pancreas systems), for which a Δt below 20 min is considered desirable^2^. Despite previous reports suggesting earlier postprandial glucose peaks in healthy adults^20–22^, earlier detection was not observed in this cohort.

Algorithm selection should therefore depend on application-specific priorities. When prioritizing sensitivity, MDA_Kölle-CGM_ offers a strong choice. For contexts requiring low FP/day while preserving moderate-to-high sensitivity, MDA_Kölle-*Ra*_ and MDA_Turksoy_ are preferable, with the latter further advantageous when training data are unavailable. If shorter detection latency is prioritized and moderate sensitivity is acceptable, MDA_Harvey_ and MDA_Dassau-2of3_ are suitable options. In contrast, MDA_Popp_, MDA_Samadi_, MDA_Faccioli_, and particularly MDA_Dassau-3of4_ appear unsuitable for use in young, healthy adults under free-living conditions.

Algorithm performance varied with dietary and anthropometric characteristics. Participants in the StandardDiet group had 84% higher odds of true-positive detection than those in the LCD group, likely reflecting the larger postprandial glucose excursions observed under higher carbohydrate intake^23^. Increased daily carbohydrate intake was also associated with higher FP/day (IRR ≈ 1.23 per 99 g increase), suggesting that prolonged glucose excursions may increase the likelihood of misclassification. In addition, higher body weight was associated with slightly reduced odds of detection (OR ≈ 0.77 per 10 kg increase in body weight). Although all participants were within a normal BMI range, this observation may be partly explained by the reported CGM bias in Abbott devices, which underestimates plasma glucose in overweight individuals^24^. These findings highlight that meal size, macronutrient composition, and interindividual physiological differences may influence CGM signal characteristics and, consequently, algorithm performance.

This study has several strengths. It is, to our knowledge, the first to directly compare nine CGM-only MDAs using a standardized holdout design under free-living conditions. Algorithm performance was evaluated across multiple metrics (sensitivity, FP/day, Δt), and mixed-effects models enabled robust comparisons accounting for day- and participant-level variability. This standardized evaluation framework supports reproducible benchmarking of CGM-only meal detection algorithms across studies. However, several limitations should be acknowledged. Meal logging adherence was inconsistent, with nearly half of all recorded days containing at least one unlogged meal, and afternoon snacks often missing. To avoid penalizing algorithms for detecting unlogged meals, afternoon periods were blinded, and a generous true-positive window was applied, likely inflating sensitivity and reducing FP/day. A wider true-positive window also increases the likelihood of matching delayed detections to logged meals. Although this may affect absolute performance estimates, the same window was applied to all algorithms and therefore does not alter the validity of the relative comparisons that were the primary focus of this study. Although this approach reduced the risk of classifying likely true but unlogged eating events as false positives, it may also have affected absolute performance estimates by suppressing valid detections. The present evaluation constraints also reduce real-world applicability, because practical implementations would not usually suppress detections during predefined daytime periods. CGM quality control allowed files with gaps of up to 120 min to maximize sample retention. Although files with longer continuous gaps were excluded, retaining shorter gaps may have increased the likelihood of FN and prolonged Δts. Algorithms were reimplemented without access to the original code, and certain logic (e.g., activation/deactivation in MDA_Samadi_) was simplified. Hyperparameter grids were restricted, and several algorithms selected values near boundary limits. The modest number of participants also limits generalizability, although repeated observations across 201 valid CGM files and 603 meal events partly strengthened the robustness of the comparative analyses. Results are limited to young, healthy adults and may not generalize to populations with diabetes, obesity, or altered glucose dynamics. Only CGM-only MDAs were included, whereas multimodal or more integrative approaches may offer performance advantages by incorporating additional physiological or behavioral signals. We nevertheless focused on CGM-only methods because they are less burdensome, less complex, and potentially more scalable for behavioral monitoring and digital nutrition applications in free-living settings.

Emerging developments in CGM and machine learning suggest that automated detection may evolve beyond meal events toward broader metabolic pattern recognition^25^. Integrating glucose-derived features with data-driven and physiological models may improve detection accuracy and support more personalized, real-time monitoring. Future work should therefore examine unified frameworks that can inform both behavioral monitoring and earlier identification of metabolic changes^26^.

This study provides a standardized comparison of CGM-only meal detection algorithms under free-living conditions and demonstrates that algorithm performance depends strongly on the relative importance of sensitivity, false detections, and detection latency. No single MDA performed best across all metrics, underscoring the need to select algorithms based on application priorities rather than expecting a universal solution. The acceptable balance between false-positive and false-negative detections is highly context dependent. In behavioral monitoring, occasional false detections can be resolved through simple participant confirmation, making higher sensitivity more valuable than strict specificity. In clinical contexts such as automated insulin delivery, however, FP must be minimized because erroneous meal detections may trigger inappropriate insulin dosing. FN also carry context-dependent implications: in behavioral or nutritional monitoring they primarily reduce completeness, whereas in clinical or intervention settings they may delay appropriate glycemic responses or impair decision support. Algorithm choice should therefore weigh the relative costs of missed versus incorrect detections. Future research should extend validation of CGM-based MDAs to more diverse populations, including individuals with type 1 or type 2 diabetes, obesity, or altered glucose dynamics. Given the observed effects of diet group and daily carbohydrate intake, meal-level macronutrient composition should be examined as a moderator of detection accuracy. More reliable food-logging protocols are needed to reduce uncertainty in ground-truth labeling. Algorithm development should prioritize reducing detection latency while maintaining moderate to high sensitivity to support just-in-time adaptive interventions. Finally, multimodal approaches that incorporate additional physiological or wearable signals may offer further gains beyond CGM-only methods.

## Methods

### Study design and participants

The present work is a secondary analysis of anonymized data derived from a 21-day randomized, controlled dietary intervention, conducted independently of the original trial objectives (ClinicalTrials.gov identifier: NCT07429058). Using these data, we evaluated CGM-based MDAs under free living conditions across three 21-day study waves at the Technical University of Munich between late 2023 and early 2024. The study protocol was approved by the ethics committee of the Technical University of Munich (approval number: 447/21 S-KH), and all participants provided written informed consent prior to participation. All methods were performed in accordance with the Declaration of Helsinki. At the explicit request of the industry sponsor, the original intervention study was not registered in a clinical trial registry.

**Fig. 2:**
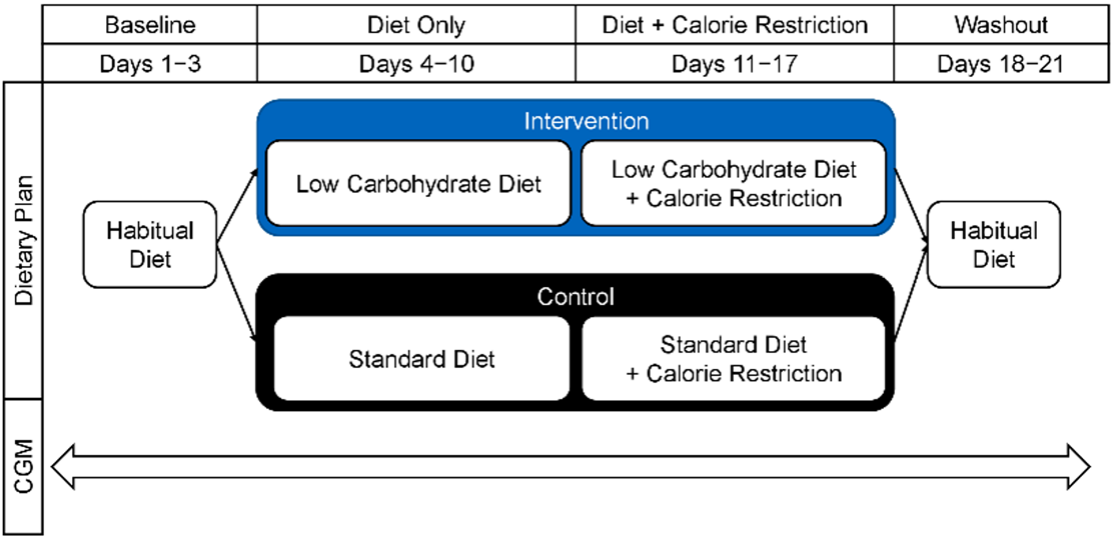
Study design of the dietary intervention.

Participants were randomized to either a low-carbohydrate diet (LCD) or a standard diet (Standard Diet), with all procedures conducted within predefined eating windows (Fig. 2). Healthy adults aged 18–40 years, without diabetes mellitus and reporting at least one day of vigorous physical activity per week, were eligible. Participants with a BMI greater than 27 kg/m^2^ or with medical conditions affecting metabolism were excluded. Of the 22 enrolled participants, six were excluded due to insufficient valid CGM data, resulting in a final sample of 16 participants (6 males, 10 females; mean age, 25.9 years; BMI, 23.4 kg/m^2^). Participant characteristics and daily carbohydrate intake by group and study phase are presented in Table 4.

**Table 4:** Participant characteristics.

	LCD	StandardDiet
Participants, *n* (%)	8 (50)	8 (50)
Sex, n (%)		
Male	4 (50)	2 (25)
Female	4 (50)	6 (75)

	Mean ± SD	Mean ± SD
Age (years)	25.6 ± 2.6	26.3 ± 4.8
Body Mass Index (kg/m^2^)	23.6 ± 2.2	23.2 ± 2.7
Daily carbohydrate intake (g)		
Baseline	335.4 ± 62.7	257.8 ± 59.1
Diet only	104.8 ± 5.9	282.8 ± 69.6
Diet + calorie restriction	104.7 ± 7.5	221.7 ± 53.1
Washout	232.4 ± 90.0	238.1 ± 84.8

*LCD* low-carbohydrate diet, *SD* standard deviation.

### Dietary intervention and schedule

The intervention included four dietary phases with standardized eating windows. All participants consumed their habitual (standard) diet during Baseline (Days 1–3). During the Diet Only period (Days 4–10), the LCD group consumed 75–125 g of carbohydrates/day, while the control group continued the standard diet. During the Diet and Caloric Restriction Period (Days 11–17), both groups adhered to their assigned diets with a daily energy deficit of ~ 500 kcal. During Washout (Days 18–21), participants returned to their habitual diet.

### CGM measurements and meal logging

Interstitial glucose was recorded at 1-min intervals using FreeStyle Libre 2 sensors (Abbott Laboratories, USA), worn on the upper arm and paired with the FreeStyle LibreLink application. Each participant used one 14-day sensor followed by one 7-day sensor to cover all 21 study days. Participants were instructed to log all meals using the Supersapiens app (TT1 Products, Inc., USA).

### Data quality control and final dataset

Each CGM file corresponded to one calendar day. Files were excluded if they contained fewer than three logged meals, had continuous gaps > 120 min, or belonged to participants with < 9 valid days. Files with shorter gaps were retained to preserve sample size and were processed together with the remaining CGM data during algorithm-specific resampling. After exclusions, 16 participants remained, contributing 201 valid CGM files and 603 meal events. The number of valid CGM files per participant ranged from 9 to 18. Further details on quality control, including file exclusion criteria, dataset composition, and the flow of participants and file inclusion and exclusion, are provided in the Supplementary Methods and Supplementary Fig. 1.

### Selection of algorithms

Candidate MDAs were identified based on Brummer et al.’s^2^ scoping review and an updated literature search in PubMed and Google Scholar (January–June 2025). From 25 CGM-based MDA candidates, we excluded those requiring additional physiological inputs^5,6,19,27–35^, lacking implementation detail^8,11,17^, or not producing explicit meal onset times^36,37^. The final set included nine reproducible and CGM-only MDAs: two voting-based detectors by Dassau et al. (MDA_Dassau-2of3_ and MDA_Dassau-3of4_)^3^, a super-twisting observer by Faccioli et al. (MDA_Faccioli_)^16^, the Glucose Rate Increase Detector (GRID) approach by Harvey et al. (MDA_Harvey_)^15^, two classifiers by Kölle et al. (MDA_Kölle-*Ra*_ and MDA_Kölle-CGM_)^13^, a simulation-based detector by Popp et al. (MDA_Popp_)^12^, a fuzzy-logic method by Samadi et al. (MDA_Samadi_)^18^, and a model-based algorithm by Turksoy et al. (MDA_Turksoy_)^14^. Full selection criteria and decision logic, including a detailed description of each MDA, are provided in the Supplementary Methods.

### Algorithm implementation

All MDAs were implemented in MATLAB R2025a (The MathWorks, Inc., USA). Each algorithm processed the preprocessed CGM time series and returned detected meal onset times. CGM files were resampled to 1-min intervals for MDA_Dassau-2of3_ and MDA_Dassau-3of4_^3^, MDA_Popp_^12^, and MDA_Turksoy_^14^, and to 5-min intervals for MDA_Faccioli_^16^, MDA_Kölle-*Ra*_ and MDA_Kölle-CGM_^13^, MDA_Harvey_^15^, and MDA_Samadi_^18^. This algorithm-specific preprocessing was chosen to preserve the intended operating conditions of each published method rather than imposing a uniform resampling scheme across all algorithms. Detailed algorithm logic, preprocessing workflows, decision rules, and mathematical formulations are provided in the Supplementary Methods, with all equations consolidated in Supplementary Table 1.

### Training, validation, testing, and performance evaluation

A per-participant holdout design was selected because the primary goal was to evaluate algorithm performance under within-individual conditions, which also enabled a fair comparison of methods that require or allow participant-specific calibration. For each participant, CGM files were pseudo-randomly split into training (31.34%; 189 meals), validation (32.84%; 198 meals), and test sets (35.82%; 216 meals). Only the classifiers by Kölle et al.^13^ were trained. Hyperparameters were tuned on the validation set by maximizing the sensitivity-focused F2-score (β = 2)^38^, consistent with de Carvalho et al.^11^ Ties were resolved by preferring lower FP/day and then shorter Δt. Final performance was assessed on the unseen test set. Detections were matched to logged meal times using a 120-min true-positive (TP) window^7,19^. TPs were detections within this window; missed meals were FN. Δt was defined as the temporal difference between detection and logged meal start. Only detections occurring after the logged meal start were included; negative values were not considered. Unmatched detections were FP. A 120-min lockout followed each TP to prevent repeated detections. Detections between 22:00 and 07:00 were excluded due to nocturnal CGM noise^3,7^. Afternoon detections (from post-lunch to 15 min before dinner) were suppressed because afternoon snacks were generally not logged, leaving the ground truth uncertain during this period. This evaluation constraint was introduced to avoid penalizing algorithms for detecting potentially true but unlogged eating events.

### Statistical analyses

For each algorithm, we calculated true positives (TP), FP, FN, sensitivity (= TP/(TP + FN)), FP/day, and Δt. Mixed-effects models were used to compare MDAs using day-level outcomes: Sensitivity was modeled with a binomial generalized linear mixed model (GLMM), FP/day with a Poisson GLMM, and Δt with a linear mixed-effects model (LMM). Fixed effects included algorithm, diet group (LCD vs. StandardDiet), phase, sex, daily carbohydrate intake, height, and weight. Participant and day were included as random effects. Continuous predictors were standardized (z-scores). Estimated marginal means (EMMs) with 95% confidence intervals (CI) were reported. Tukey-adjusted pairwise comparisons used MDA_Kölle-CGM_ (Supplementary Methods and Supplementary Table 1) as the reference, with *p* < 0.05 considered significant. Effect sizes were expressed as odds ratios (OR), incidence rate ratios (IRR), or standardized β coefficients. Analyses were conducted in RStudio 2025.05.1 (Posit Software, USA).

## Supplementary Information

Below is the link to the electronic supplementary material.

Supplementary Material 1.

## Data Availability

Data will be available from the corresponding author upon reasonable request.

## References

[1] Höchsmann, C. & Martin, C. K. Review of the validity and feasibility of image-assisted methods for dietary assessment. *Int. J. Obes.* **44**, 2358–2371. 10.1038/s41366-020-00693-2 (2020). 10.1038/s41366-020-00693-2 PMC7686022 33033394

[2] Brummer, J., Glasbrenner, C., Hechenbichler Figueroa, S., Koehler, K. & Höchsmann, C. Continuous glucose monitoring for automatic real-time assessment of eating events and nutrition: a scoping review. *Front. Nutr.* **10**, 1308348. 10.3389/fnut.2023.1308348 (2023). 38264192 10.3389/fnut.2023.1308348 PMC10804456

[3] Dassau, E., Bequette, B. W., Buckingham, B. A. & Doyle, F. J. Detection of a meal using continuous glucose monitoring: implications for an artificial beta-cell. *Diabetes Care*. **31**, 295–300. 10.2337/dc07-1293 (2008). 17977934 10.2337/dc07-1293

[4] ElSayed, N. A. et al. 6. Glycemic targets: standards of care in diabetes-2023. *Diabetes Care*. **46**, S97–S110. (2023). 10.2337/dc23-S006 36507646 10.2337/dc23-S006 PMC9810469

[5] Fathi, A. E., Palisaitis, E., Boulet, B., Legault, L. & Haidar, A. An unannounced meal detection module for artificial pancreas control systems. in *American Control Conference (ACC)* 4130–4135. 10.23919/ACC.2019.8814932 (2019).

[6] Godoy, J. L., Sereno, J. E. & Rivadeneira, P. S. Meal detection and carbohydrate estimation based on a feedback scheme with application to the artificial pancreas. *Biomed. Signal. Process. Control*. **68**, 102715. 10.1016/j.bspc.2021.102715 (2021).

[7] Ornetzeder, C. et al. Feasibility of fully closed loop insulin delivery in type 2 diabetes. *2019 IEEE Conference Control Technology and Application. (CCTA)*. 906–913 10.1109/CCTA.2019.8920591 (2019).

[8] Palisaitis, E., El Fathi, A., von Oettingen, J. E., Haidar, A. & Legault, L. A meal detection algorithm for the artificial pancreas: a randomized controlled clinical trial in adolescents with type 1 diabetes. *Diabetes Care*. **44**, 604–606. 10.2337/dc20-1232 (2021). 33277302 10.2337/dc20-1232

[9] Turksoy, K., Quinn, L. T., Littlejohn, E. & Cinar, A. An integrated multivariable artificial pancreas control system. *J. Diabetes Sci. Technol.* **8**, 498–507. 10.1177/1932296814524862 (2014). 24876613 10.1177/1932296814524862 PMC4455451

[10] Holzer, R., Bloch, W. & Brinkmann, C. Continuous glucose monitoring in healthy adults-possible applications in health care, wellness, and sports. *Sensors* **22**, 2030. 10.3390/s22052030 (2022). 35271177 10.3390/s22052030 PMC8915088

[11] de Carvalho, D., Kaymak, U. & Van Gorp, P. Riel, N. Data-driven meal events detection using blood glucose response patterns. *BMC Med. Inf. Decis. Mak.* **23**, 282. 10.1186/s12911-023-02380-4 (2023). van. 10.1186/s12911-023-02380-4 PMC10709931 38066494

[12] Popp, C. J. et al. Objective determination of eating occasion timing: combining self-report, wrist motion, and continuous glucose monitoring to detect eating occasions in adults with prediabetes and obesity. *J. Diabetes Sci. Technol.* **18**, 266–272. 10.1177/19322968231197205 (2024). 37747075 10.1177/19322968231197205 PMC10973869

[13] Kölle, K., Biester, T., Christiansen, S., Fougner, A. L. & Stavdahl, O. Pattern recognition reveals characteristic postprandial glucose changes: non-individualized meal detection in diabetes mellitus type 1. *IEEE J. Biomed. Health Inf.* **24**, 594–602. 10.1109/JBHI.2019.2908897 (2020). 10.1109/JBHI.2019.2908897 30951481

[14] Turksoy, K. et al. Meal detection in patients with type 1 diabetes: a new module for the multivariable adaptive artificial pancreas control system. *IEEE J. Biomed. Health Inf.* **20**, 47–54. 10.1109/JBHI.2015.2446413 (2016). 10.1109/JBHI.2015.2446413 PMC4713125 26087510

[15] Harvey, R. A., Dassau, E., Zisser, H., Seborg, D. E. & DoyleIII, F. J. Design of the glucose rate increase detector: a meal detection module for the health monitoring system. *J. Diabetes Sci. Technol.* **8**, 307–320. 10.1177/1932296814523881 (2014). 24876583 10.1177/1932296814523881 PMC4455414

[16] Faccioli, S. et al. Super-twisting-based meal detector for type 1 diabetes management: improvement and assessment in a real-life scenario. *Comput. Methods Programs Biomed.* **219**, 106736. 10.1016/j.cmpb.2022.106736 (2022). 35338888 10.1016/j.cmpb.2022.106736

[17] Fushimi, E., Colmegna, P., De Battista, H., Garelli, F. & Sánchez-Peña, R. Unannounced meal analysis of the ARG algorithm. in. *American Control Conference (ACC)* 4740–4745. 10.23919/ACC.2019.8814719 (2019).

[18] Samadi, S. et al. Automatic detection and estimation of unannounced meals for multivariable artificial pancreas system. *Diabetes Technol. Ther.* **20**, 235–246. 10.1089/dia.2017.0364 (2018). 29406789 10.1089/dia.2017.0364 PMC5867514

[19] Weimer, J., Chen, S., Peleckis, A., Rickels, M. R. & Lee, I. Physiology-invariant meal detection for type 1 diabetes. *Diabetes Technol. Ther.* **18**, 616–624. 10.1089/dia.2015.0266 (2016). 27704875 10.1089/dia.2015.0266 PMC6528748

[20] Daenen, S. et al. Peak-time determination of post-meal glucose excursions in insulin-treated diabetic patients. *Diabetes Metab.* **36**, 165–169. 10.1016/j.diabet.2009.12.002 (2010). 20226708 10.1016/j.diabet.2009.12.002

[21] Freckmann, G. et al. Continuous glucose profiles in healthy subjects under everyday life conditions and after different meals. *J. Diabetes Sci. Technol.* **1**, 695–703. 10.1177/193229680700100513 (2007). 19885137 10.1177/193229680700100513 PMC2769652

[22] Hijikata, M., Higa, M., Ichijo, T. & Hirose, T. A comparison of meal tolerance test and oral glucose tolerance test for predicting insulin therapy in patients with gestational diabetes. *Food Nutr. Res.*10.29219/fnr.v65.5490 (2021). 33776619 10.29219/fnr.v65.5490 PMC7955519

[23] Laivina, E., Figueroa, S. H., Duni, D., Amaya, J. & Koehler, K. Acute differential effects of low-carbohydrate versus standard diet during caloric maintenance and moderate caloric deficit on postprandial glucose response among young healthy adults. in *Adipositas—Ursachen, Folgeerkrankungen, Therapie* vol. 18 KP2-7Georg Thieme Verlag KG. 10.1055/s-0044-1788855 (2024).

[24] Ghane, N. et al. Estimating plasma glucose with the FreeStyle Libre Pro continuous glucose monitor during oral glucose tolerance tests in youth without diabetes. *Pediatr. Diabetes*. **20**, 1072–1079. 10.1111/pedi.12910 (2019). 31433542 10.1111/pedi.12910 PMC6821586

[25] Montaser, E., Sosenko, J. M. & Ismail, H. M. Predicting the timing of the metabolic inflection point in type 1 diabetes progression using machine learning and survival analysis models. *Diabetes*10.2337/db25-0961 (2026). 41860454 10.2337/db25-0961 PMC13110513

[26] Mader, J. K. et al. The use of continuous glucose monitoring to diagnose stage 2 type 1 diabetes. *J. Diabetes Sci. Technol.* **19**, 1109–1127. 10.1177/19322968251333441 (2025). 40444471 10.1177/19322968251333441 PMC12125016

[27] Atlas, E., Nimri, R., Miller, S., Grunberg, E. A. & Phillip, M. MD-logic artificial pancreas system: a pilot study in adults with type 1 diabetes. *Diabetes Care*. **33**, 1072–1076. 10.2337/dc09-1830 (2010). 20150292 10.2337/dc09-1830 PMC2858178

[28] Mosquera-Lopez, C. et al. Enabling fully automated insulin delivery through meal detection and size estimation using Artificial Intelligence. *Npj Digit. Med.* **6**, 1–7. 10.1038/s41746-023-00783-1 (2023). 36914699 10.1038/s41746-023-00783-1 PMC10011368

[29] Ibrahim, M., Beneyto, A., Contreras, I. & Vehi, J. An ensemble machine learning approach for the detection of unannounced meals to enhance postprandial glucose control. *Comput. Biol. Med.* **171**, 108154. 10.1016/j.compbiomed.2024.108154 (2024). 38382387 10.1016/j.compbiomed.2024.108154

[30] Ramkissoon, C. M., Herrero, P., Bondia, J. & Vehi, J. Unannounced meals in the artificial pancreas: detection using continuous glucose monitoring. *Sensors* **18**, 884. 10.3390/s18030884 (2018). 29547553 10.3390/s18030884 PMC5876595

[31] Dovc, K. et al. Faster Compared With Standard Insulin Aspart During Day-and-Night Fully Closed-Loop Insulin Therapy in Type 1 Diabetes: A Double-Blind Randomized Crossover Trial. *Diabetes Care*. **43**, 29–36. 10.2337/dc19-0895 (2020). 31575640 10.2337/dc19-0895

[32] Bertrand, L., Cleyet-Marrel, N. & Liang, Z. Recognizing eating activities in free-living environment using consumer wearable devices. *Eng. Proc.* **6**, 58. (2021). 10.3390/I3S2021Dresden-10141

[33] Palacios, V., Woodbridge, D. M. K. & Fry, J. L. Machine learning-based meal detection using continuous glucose monitoring on healthy participants: an objective measure of participant compliance to protocol. in *Annual International Conference of the IEEE Engineering in Medicine and Biology Society* 7032–7035. 10.1109/EMBC46164.2021.9630408 (2021). 10.1109/EMBC46164.2021.9630408 34892722

[34] Bertrand, L., Cleyet-Marrel, N. & Liang, Z. The role of continuous glucose monitoring in automatic detection of eating activities. in *IEEE 3rd Global Conference on Life Sciences and Technologies (LifeTech)* 313–314. 10.1109/LifeTech52111.2021.9391849 (2021).

[35] Presseller, E. K., Parker, M. N., Zhang, F., Manasse, S. & Juarascio, A. S. Continuous glucose monitoring as an objective measure of meal consumption in individuals with binge-spectrum eating disorders: a proof-of-concept study. *Eur. Eat. Disord Rev. J. Eat. Disord Assoc.* **32**, 828–837. 10.1002/erv.3094 (2024). 10.1002/erv.3094 PMC11282580 38568882

[36] Pellizzari, E. et al. Automatic identification of unreported meals from continuous glucose monitoring data in individuals after bariatric surgery using a template matching algorithm. *Sci. Rep.* **15**, 7797. 10.1038/s41598-025-92275-3 (2025). 40050410 10.1038/s41598-025-92275-3 PMC11885432

[37] Hoyos, J. D. et al. Characterization of glycemic patterns in type 1 diabetes without insulin or meal input data. in. *10th International Conference on Systems and Control (ICSC)* 576–581. 10.1109/ICSC57768.2022.9993837 (2022).

[38] Sokolova, M., Japkowicz, N. & Szpakowicz, S. *Beyond Accuracy, F-Score and ROC: A Family of Discriminant Measures for Performance Evaluation*. *AI 2006: Advances in Artificial Intelligence, Lecture Notes in Computer Science* vol. Vol. 4304 1021 ISBN: 978-3-540-49787-5 (2006).

