# Supplementary material for: Performance of continuous glucose monitoring-based meal detection algorithms in young healthy adults
Source: Sci Rep. 2026 May 19;16:15714. doi: 10.1038/s41598-026-50699-5 (PMC13190723; doi:10.1038/s41598-026-50699-5)
Supplement: Supplementary file 1 — Supplementary Material 1. [file 41598_2026_50699_MOESM1_ESM.pdf]

SUPPLEMENTARY MATERIAL

TITLE: Performance of continuous glucose monitoring-based meal detection algorithms in young healthy adults

AUTHORS: Christoph Höchsmann, Jonas T Weber, Sieglinde Hechenbichler Figueroa, Elizabete Laivina, Karsten Koehler

TABLE OF CONTENTS

Supplementary Methods 2
Data Quality Control Details 2
Supplementary Figure 1. 2
Algorithm Selection Criteria and Literature Screening Details 3
Detailed Algorithm Logic and Assumptions 3
Algorithms by Dassau et al. 3
Algorithm by Faccioli et al. 4
Algorithm by Harvey et al. 4
Algorithms by Kölle et al. 5
Algorithm by Popp et al. 6
Algorithm by Samadi et al. 6
Algorithm by Turksoy et al. 7
Supplementary Table 1. 8
Supplementary Results 11
Supplementary Table 2. 11
Supplementary Table 3. 12
References 15

SUPPLEMENTARY METHODS

Data Quality Control Details

Each continuous glucose monitoring (CGM) file represented one calendar day. To ensure completeness and consistency of data, files were excluded according to the following sequential criteria:

(1) Files containing fewer than three logged meals (breakfast, lunch, dinner).

(2) Files with continuous CGM gaps exceeding 120 minutes.

(3) Participants for whom fewer than nine valid CGM files remained after applying criteria 1 and 2.

Six participants were excluded due to insufficient valid CGM data. A total of 217 CGM files were excluded under criterion 1 and 22 files under criterion 2. The final dataset consisted of 201 valid CGM files from 16 participants, corresponding to 603 meal events. Across all included files, 204,369 observed CGM samples were available out of 289,440 expected samples, resulting in 70.6% data completeness. The mean (standard deviation [SD]) glucose concentration across all included samples was 97.9 (17.8) mg·dL^{-1}.

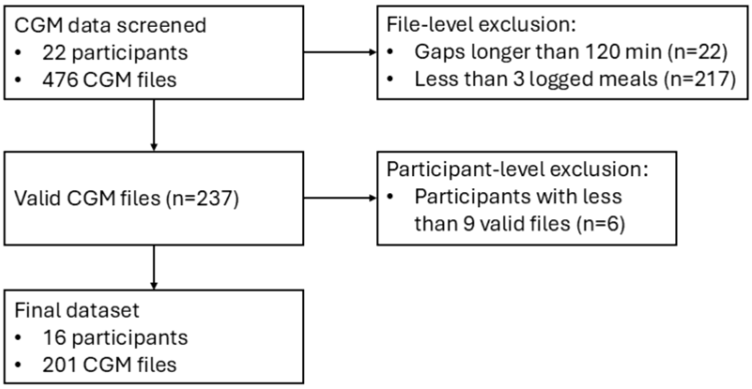

Supplementary Figure 1. Flow diagram of data inclusion and exclusion.

Algorithm Selection Criteria and Literature Screening Details

Brummer et al.'s [1] scoping review on CGM-based automatic assessment of eating events served as the basis for algorithm identification. A supplementary literature search was conducted using PubMed and Google Scholar. The initial search was conducted in January 2025 and was updated through June 23, 2025. Publications were retained if the algorithms were validated in human participants and reported performance metrics were provided. Across the included literature, some publications introduced more than one MDA,[2–4] while others, such as Ornetzeder et al.[5], did not present a novel algorithm. In total, 25 candidate MDAs were identified. Algorithms were excluded if they:

(1) lacked essential implementation details, preventing faithful reproduction,[6–8]
(2) required additional inputs beyond CGM time series (e.g., insulin pump data),[9–15]
(3) depended on additional physiological or physical activity data,[16–18]
(4) required anthropometric data or clinical diagnoses,[19] or
(5) did not determine exact meal detection times. [20,21]

After applying these exclusion criteria, nine MDAs were retained for implementation and validation.

Detailed Algorithm Logic and Assumptions

Algorithms by Dassau et al.

The two MDAs developed by Dassau et al.[4] were originally designed to detect upcoming meals in artificial pancreas systems. Both methods calculate glucose rise indicators at each 1-minute CGM sample i, derived from raw or Kalman-filtered glucose values. Four indicators are computed:

(1) a three-point backward-difference rate of change (ROC) using raw glucose BD_{raw} (BD, *Equation 1*, **Supplementary Table 1**),
(2) a three-point backward-difference ROC using Kalman-filtered glucose BD_{Kalman} (BDK, *Equation 2*, **Supplementary Table 1**),

(3) Kalman-based estimates of glucose and its ROC (KF, *Equation 3*, **Supplementary Table 1**), and

(4) the acceleration of the Kalman estimate (ACC, *Equation 4*, **Supplementary Table 1**).

In the two-of-three-voting detector ($MDA_{Dassau\text{-}2of3}$), a meal is declared when at least two of three indicators (BD, BDK, ACC) are true within a 5-minute window of consecutive samples (i, …, i + 4), as shown in *Equation 5* (**Supplementary Table 1**). In the three-of-four-voting detector ($MDA_{Dassau\text{-}3of4}$), a meal is declared when any three of the four indicators are simultaneously true throughout the same 5-minute window (*Equation 6*, **Supplementary Table 1**). Both algorithms were implemented at a 1-minute sampling frequency in this study. Thresholds were tuned per participant based on the performance of the validation set.

Algorithm by Faccioli et al.

The MDA proposed by Faccioli et al.[22] was developed to improve postprandial detection in automated insulin delivery systems by reducing false alarms. A super-twisting observer generates a residual signal $Res(i)$ at each CGM sample i, highlighting unexpected rises in glucose, such as those following meal intake. In parallel, the glucose derivative $\hat{\dot{G}}(i)$ is estimated via a Kalman filter. A meal is declared when both signals exceed their respective participant-specific tuned thresholds, as shown in *Equation 7* (**Supplementary Table 1**):

$$\hat{\dot{G}}(i) > Th_{Der} \text{ and } Res(i) > Th_{Res}$$

In contrast to the original implementation, observer gain was tuned separately for each day, and a 3-point median filter was applied to reduce CGM noise before processing. Thresholds were optimized individually per participant using the validation set.

Algorithm by Harvey et al.

The Glucose Rate Increase Detector (GRID) by Harvey et al.[23] was designed to rapidly identify postprandial glucose excursions. First, the CGM signal is passed through a noise-spike filter and a low-pass filter with time constant τF. The ROC of the filtered signal,

denoted G'_F, is then computed using the derivative of a 3-point Lagrange interpolation polynomial at each CGM sample i. A meal is detected when the filtered glucose concentration $G_F(i)$ exceeds a minimum threshold G_{min}, and either:

- the last three ROC values exceed threshold $G'_{min,3}$, or
- the last two exceed threshold $G'_{min,2}$.

This logic is formalized in *Equation 8* (**Supplementary Table 1**). All four hyperparameters (τF, G_{min}, $G'_{min,3}$, $G'_{min,2}$) were tuned individually for each participant using validation set performance.

Algorithms by Kölle et al.

<u>Classification of Estimated Ra Horizons (MDA$_{\text{Kölle-}Ra}$)</u>

Kölle et al.[3] proposed a classifier-based MDA that detects meals using estimated glucose appearance rates (R_a). The CGM signal is first smoothed via a Kalman filter, after which R_a (i) is estimated based on a Bergman minimal model.[24] At each 5-minute CGM sample i, a 100-minute horizon is constructed from the most recent 20 values of the estimated R_a trajectory. Each horizon is labeled as a meal onset if a meal was logged within the preceding 60 minutes; otherwise, it is labeled as non-meal. A linear discriminant analysis (LDA) classifier is then trained on these features. At test time, a meal is declared when the posterior probability of meal onset $\rho_{LDA}(meal \mid \cdot)$ given the features X_i (R_a) (100-min CGM horizon [$R_a(i$−19),...,$R_a(i)$]) exceeds a selected threshold θ_{post} on the current 5-min CGM sample i (*Equation 9*, **Supplementary Table 1**).

<u>Classification of CGM Horizons (MDA$_{\text{Kölle-CGM}}$)</u>

In this variant, the CGM signal is again smoothed via a Kalman filter, but the 100-minute feature horizons are constructed directly from the CGM values rather than estimated R_a. The labeling procedure and LDA classifier training are identical to those of MDA$_{\text{Kölle-}Ra}$. A meal is detected when the posterior probability ρ_{LDA} ($meal \mid \cdot$) given the features $X_i(G)$ (100-min CGM horizon [$G(i$−19),...,$G(i)$]) exceeds threshold θ_{post} on the current 5-min CGM sample i

(*Equation 10*, **Supplementary Table 1**).

Tuning was conducted using the validation dataset, and model parameters were tuned over the following ranges: $\delta \in \{10^{-15:3:3}\}$, $\gamma \in \{0, 0.05, ..., 1\}$, and $\theta_{post} \in \{0.05, 0.10, ..., 0.95\}$.

Algorithm by Popp et al.

Popp et al.[2] proposed a simulation-based algorithm that detects meal onsets by identifying when a physiological glucose-insulin model best explains deviations between observed and model-predicted CGM values. The method uses the Dalla Man minimal model to simulate expected glucose trajectories, assuming no meal intake.[25] At each time point i, the divergence score Div is computed as the mean relative error between observed CGM values $G(i)$ and model predictions $G'(i)$ over a 30-minute window ζ, as shown in *Equation 11* (**Supplementary Table 1**). When this divergence exceeds a threshold ϕ, the time point is flagged as a candidate meal onset. For each candidate, the algorithm back-searches up to a maximum delay τ to locate the earliest divergence, which is treated as the meal start m_{st}. Around each candidate meal, glucose concentration is resimulated using discrete carbohydrate loads of 25 g, 50 g, 75 g, and 100 g, assuming a 90-minute meal duration. For each simulated load, a fit error $D(m)$ is calculated as the Euclidean distance between simulated and observed glucose within the evaluation window $[m_{st}, i]$ (*Equation 12*, **Supplementary Table 1**). A meal flag is raised when the fit error for a given carbohydrate load falls below the error threshold ε, as formalized in *Equation 13* (**Supplementary Table 1**). This method does not require participant-specific tuning. Global parameters were defined as:

ζ = 30 min, $\phi \in \{10, 12\}$, $\tau \in \{20, 30, 40, 50, 60\}$ min, and $\varepsilon \in \{14, 15, 16, 17, 18\}$

The grid was kept small due to high computational demand.

Algorithm by Samadi et al.

The fuzzy-logic detector developed by Samadi et al.[26] identifies unannounced meals from CGM time series in artificial pancreas applications. At each 5-minute CGM sample i, a

quadratic polynomial is fit to the four most recent glucose points, and the first and second derivatives ($d_1G(i)$,$d_2G(i)$) are computed. The first derivative is assigned fuzzy memberships across negative, zero, and positive values based on a slope gain parameter γ_1. The second derivative is used to determine whether the trend is accelerating or decelerating. These memberships form seven qualitative glucose shape categories (B, F, C, G, A, E, D), ranging from strongly decreasing to strongly increasing. An increase-of-glucose trend (IGT) score is computed as a weighted average of these memberships, as shown in *Equation 14* (**Supplementary Table 1**). In this study, we applied a simplified detection rule by omitting the activation, pause, and deactivation logic described by Samadi et al. [26] and instead raised a meal flag when $IGT(i)$ within [−3,3] exceeded the activation threshold $Threshold_{act}$, as shown in *Equation 15* (**Supplementary Table 1**). The slope gain $\gamma_1 \in \{1.00,1.50,...,4.00\}$ and $Threshold_{act} \in \{1.0,1.1,...,3.0\}$ were tuned individually per participant.

Algorithm by Turksoy et al.

The MDA proposed by Turksoy et al.[27] was developed to detect unannounced meals in artificial pancreas settings using a modified Bergman minimal model with a two-compartment meal subsystem. The state vector at each 1-minute CGM sample i includes: 1) effective insulin I_{eff}, 2) subcutaneous glucose concentration G_s, 3) current and lagged glucose appearance rates (R_a, $R_a(i-1)$), 4) physiological parameters p_1, p_2, p_4, and 5) a meal time constant τ. An Unscented Kalman Filter (UKF) is used to estimate the state vector $\hat{x}(i)$, including the estimated rate of glucose $\hat{R}_a(i)$. A meal is detected when $\hat{R}_a(i)$ exceeds a threshold $Threshold_{Ra}$, while glucose concentration G_s is above 100 mg/dL, as defined in *Equation 16* (**Supplementary Table 1**). Parameter $Threshold_{Ra}$ was tuned for each participant over the range 1.5–2.5 mg/dL/min. Unlike the original implementation, the post-detection threshold raising was not applied; instead, a detection lockout was enforced, as described in the main **Methods (Training, Validation, Testing)**.

Supplementary Table 1. Mathematical definitions of meal detection algorithms.

Equation No.	Algorithm	Description / Purpose	Equation
Equation 1	Dassau et al.[4]	Indicator based on backward-difference on raw glucose BD_{raw} (BD)	$$\mathrm{BD}(i) = \begin{cases} 1, \text{ if } \mathrm{BD}_{\mathrm{raw}}(i) > \mathrm{Threshold}_{\mathrm{ROC}} \\ 0, \text{ otherwise} \end{cases}$$ where $\mathrm{Threshold}_{\mathrm{ROC}} \in \{1.0, 1.1, 1.2, \ldots, 3.0\}$ mg/dL/min
Equation 2	Dassau et al.[4]	Indicator based on backward-difference on Kalman-filtered glucose BD_{Kalman} (BDK)	$$\mathrm{BDK}(i) = \begin{cases} 1, \text{ if } \mathrm{BD}_{\mathrm{Kalman}}(i) > \mathrm{Threshold}_{\mathrm{ROC}} \\ 0, \text{ otherwise} \end{cases}$$ where $\mathrm{Threshold}_{\mathrm{ROC}} \in \{1.0, 1.1, 1.2, \ldots, 3.0\}$ mg/dL/min
Equation 3	Dassau et al.[4]	Kalman estimates of glucose and rate of change indicator (KF)	$$\mathrm{KF}(i) = \begin{cases} 1, \text{ if } G'_{Kalman}(i) > \mathrm{Threshold}_{\mathrm{ROC}} \\ \quad \wedge\ G'_{Kalman}(i) < \mathrm{Threshold}_{\mathrm{maxROC}} \\ \quad \wedge\ G_{Kalman}(i) > \mathrm{Threshold}_{\mathrm{Glucose}} \\ 0, \text{ otherwise} \end{cases}$$ where $\mathrm{Threshold}_{\mathrm{ROC}} \in \{1.0, 1.1, 1.2, \ldots, 3.0\}$ mg/dL/min, $\mathrm{Threshold}_{\mathrm{maxROC}} \in \{2, 3, 4, 5\}$ mg/dL/min $\mathrm{Threshold}_{\mathrm{Glucose}} \in \{100, 110, \ldots, 200\}$ mg/dL/min
Equation 4	Dassau et al.[4]	Acceleration of Kalman estimate indicator (ACC)	$$\mathrm{ACC}(i) = \begin{cases} 1, \text{ if } G''_{Kalman}(i) > \mathrm{Threshold}_{\mathrm{Acceleration}} \\ 0, \text{ otherwise} \end{cases}$$ where $\mathrm{Threshold}_{\mathrm{Acceleration}} \in \{0.2, 0.3, \ldots, 0.8\}$ mg/dL/min^2
Equation 5	Dassau et al.[4]	Two of three voting rule across BD, BDK, ACC ($MDA_{Dassau\text{-}2of3}$)	$$\mathrm{MDA}_{\mathrm{Dassau\_2of3}}(i) = \begin{cases} 1, \text{ if } \sum_{j=0}^{4} 1\{\mathrm{BD}(i+j) + \mathrm{BDK}(i+j) + \mathrm{ACC}(i+j) \geq 2\} = 5 \\ 0, \text{ otherwise} \end{cases}$$

Equation 6	Dassau et al.[4]	Three of four voting rule across BD, BDK, KF, ACC ($MDA_{Dassau\text{-}3of4}$)	$\mathrm{MDA}_{\mathrm{Dassau\_3of4}}(i) = \begin{cases} 1, \text{ if } \sum_{j=0}^{4} 1\{\mathrm{BD}\,(i+j) + \mathrm{BDK}\,(i+j) + \mathrm{KF}\,(i+j) + \mathrm{ACC}\,(i+j) \geq 3\} = 5 \\ 0, \text{ otherwise} \end{cases}$
Equation 7	Faccioli et al.[22]	Observer residual plus derivative threshold rule ($MDA_{Faccioli}$)	$\mathrm{MDA}_{\mathrm{Faccioli}}(i) = \begin{cases} 1, \text{ if } Res(i) > Th_{Res} \wedge \widehat{\dot{G}}(i) > Th_{Der} \\ 0, \text{ otherwise} \end{cases}$<br>where $Th_{Res} \in \{1.0, 1.1, \ldots, 4.0\}$ mg/dL<br>$Th_{Der} \in \{0.2, 0.3, 0.4, 0.5\}$ mg/dL/min
Equation 8	Harvey et al.[23]	GRID: filtered glucose plus rate of change thresholds (MDA_{Harvey})	$\mathrm{MDA}_{\mathrm{Harvey}}(i) = \begin{cases} 1, \text{ if } G_F(i) > G_{min} \wedge (G'_F(i-2{:}i) > G'_{min,3} \vee G'_F(i-1{:}i) > G'_{min,2}) \\ 0, \text{ otherwise} \end{cases}$<br>where $G_{min} \in \{100, 105, \ldots, 140\}$ mg/dL<br>$G'_{min,3} \in \{0.8, 0.9, \ldots, 1.8\}$ mg/dL/min<br>$G'_{min,2} \in \{0.8, 0.9, \ldots, 1.8\}$ mg/dL/min<br>$\tau F \in \{4, 5, 6, 7, 8\}$ min
Equation 9	Kölle et al.[3]	LDA posterior threshold on R_α-based horizons ($MDA_{Kölle\text{-}Ra}$)	$\mathrm{MDA}_{\text{Kölle-}Ra}(i) = 1\{\rho_{LDA}(\mathrm{meal} \mid X_i(R_a)) \geq \theta_{post}\}$
Equation 10	Kölle et al.[3]	LDA posterior threshold on CGM-based horizons ($MDA_{Kölle\text{-}CGM}$)	$\mathrm{MDA}_{\text{Kölle-CGM}}(i) = 1\{\rho_{LDA}(\mathrm{meal} \mid X_i(G)) \geq \theta_{post}\}$
Equation 11	Popp et al.[2]	Divergence score definition	$\mathrm{Div}(i) = \frac{1}{\zeta} \sum_{j=0}^{\zeta-1} \left\| \frac{G(i-j) - G'(i-j)}{G'(i-j)} \right\|$
Equation 12	Popp et al.[2]	Fit error (Euclidean distance) definition	$\mathrm{D(m)} = \sqrt{\frac{1}{n} \sum_{k=k_0}^{k_1} \left(G'_{\mathrm{m}}(\mathrm{k}) - G(\mathrm{k})\right)^2}$

Equation 13	Popp et al.[2]	Meal detection decision rule (divergence plus fit) (MDA_{Popp})	$\mathrm{MDA_{Popp}}(i) = \begin{cases} 1, \text{ if } \mathrm{Div}(i) > \phi \text{ and } \exists\, m \in M{:}\ \mathrm{D}(m) < \varepsilon \\ 0, \text{ otherwise} \end{cases}$
Equation 14	Samadi et al.[26]	Increase of glucose trend (IGT) definition	$\mathrm{IGT}(i) = \frac{-3\mathrm{B(i)} - 2\mathrm{F(i)} - \mathrm{C(i)} + \mathrm{A(i)} + 2\mathrm{E(i)} + 3\mathrm{D(i)}}{\mathrm{A(i)} + \mathrm{B(i)} + \mathrm{C(i)} + \mathrm{D(i)} + \mathrm{E(i)} + \mathrm{F(i)} + \mathrm{G(i)}}$
Equation 15	Samadi et al.[26]	IGT activation threshold rule (MDA_{Samadi})	$\mathrm{MDA_{Samadi}}(i) = \begin{cases} 1, \text{ if } \mathrm{IGT}(i) > \mathrm{Threshold_{act}} \\ 0, \text{ otherwise} \end{cases}$
Equation 16	Turksoy et al.[27]	R_a threshold crossing with glucose constraint ($MDA_{Turksoy}$)	$\mathrm{MDA_{Turksoy}}(i) = \begin{cases} 1, \text{ if } \hat{R}_a(i) > \mathrm{Threshold}_{R_a} \wedge\ G_s(i) > 100 \text{ mg/dL} \\ 0, \text{ otherwise} \end{cases}$

Abbreviations: CGM, continuous glucose monitoring; GRID, glucose rate increase detector; LDA, linear discriminant analysis.

SUPPLEMENTARY RESULTS

Supplementary Table 2. Comparison of algorithm performance with original publications.

MDA	Population, Setting	TP Window	Detection Lockout	Published Metrics	Metrics (This Work)
$MDA_{Dassau\text{-}2of3}$ [4]	T1DM pediatric, inpatient	Not reported	Post-meal: 15-20 min & night lockout	Sensitivity: not reported<br>FP/day: not reported<br>Δt: 30 min	Sensitivity: 72.2%<br>FP/day 0.6<br>Δt: 37.6 min
$MDA_{Dassau\text{-}3of4}$ [4]	T1DM pediatric, inpatient	Not reported	After meal: 15–20 min & night lockout	Sensitivity: not reported<br>FP/day: not reported<br>Δt: 32 min	Sensitivity: 49.1%<br>FP/day 0.12<br>Δt: 36.8 min
$MDA_{Faccioli}$ [22]	T1DM adults, free-living	Not reported	Not reported	Sensitivity: 70.0% (13) [a]<br>FP/day: 1.4 (1.4)<br>Δt: 45 (45) min	Sensitivity: 64.4%<br>FP/day: 1.39<br>Δt: 39.6 min
MDA_{Harvey} [23] (metrics from Ornetzeder et al. [5] and Kölle et al.[3])	T2DM older adults/ T1DM pediatric, free-living	120 min/ 60 min	After meal: 120 min / 30 min	Sensitivity: 79.9/ 21.0%<br>FP/day: 1.0/ 2.81<br>Δt: 30.7/42.5 min	Sensitivity: 70.4%<br>FP/day: 0.26<br>Δt: 37.3 min
$MDA_{Kölle\text{-}Ra}$ [3]	T1DM pediatric, free-living	60 min	After meal: 30 min	Sensitivity: 90%<br>FP/day: 1.50<br>Δt: 18.6 min	Sensitivity: 77.3%<br>FP/day: 0.33<br>Δt: 41.7 min
$MDA_{Kölle\text{-}CGM}$ [3]	T1DM pediatric, free-living	60 min	After meal: 30 min	Sensitivity: 92%<br>FP/day: 1.37<br>Δt: 32.7 min	Sensitivity: 82.4%<br>FP/day: 0.39<br>Δt: 43.8 min
MDA_{Popp} [2]	T2DM & prediabetes adults, free-living	Not reported	Not reported	Sensitivity: not reported<br>FP/day: not reported<br>Δt: not reported	Sensitivity: 82.9%<br>FP/day: 1.28<br>Δt: 60.5 min
MDA_{Samadi} [26]	T1DM adults, not reported	120 min	No discrete lockout	Sensitivity: 94% (meals), 68% (snacks)<br>FP/day: 1.05; Δt: 34.8 min	Sensitivity: 89.8%<br>FP/day: 2.42<br>Δt: 58.5 min
$MDA_{Turksoy}$ [27] (metrics from Kölle et al.[3])	T1DM pediatric, inpatient	−30 to 120 min	No discrete lockout	Sensitivity: 97/64%<br>FP/day: 1.28<br>Δt: 32.67 min	Sensitivity: 76.9%<br>FP/day: 0.22<br>Δt: 40.7 min

[a] Data are Median (IQR)

Abbreviations: IQR, interquartile range; MDA, meal detection algorithm; FP/day, false positives per day; Δt, detection time; TP window, true-positive window; T1DM, type 1 diabetes mellitus; T2DM, type 2 diabetes mellitus

Supplementary Table 3. Pairwise comparisons of sensitivity, FP/day, and Δt.

Sensitivity

Contrast	Odds Ratio	SE	P value
$MDA_{Kölle\text{-}CGM}$ / $MDA_{Dassau\text{-}2of3}$	1.93	0.47	0.1596
$MDA_{Kölle\text{-}CGM}$ / $MDA_{Dassau\text{-}3of4}$	6.01	1.44	<.0001
$MDA_{Kölle\text{-}CGM}$ / $MDA_{Faccioli}$	2.92	0.70	0.0003
$MDA_{Kölle\text{-}CGM}$ / MDA_{Harvey}	2.14	0.52	0.0495
$MDA_{Kölle\text{-}CGM}$ / $MDA_{Kölle\text{-}Ra}$	1.43	0.36	0.9001
$MDA_{Kölle\text{-}CGM}$ / MDA_{Popp}	0.97	0.26	1
$MDA_{Kölle\text{-}CGM}$ / MDA_{Samadi}	0.50	0.15	0.3362
$MDA_{Kölle\text{-}CGM}$ / $MDA_{Turksoy}$	1.47	0.37	0.8488
$MDA_{Dassau\text{-}2of3}$ / $MDA_{Dassau\text{-}3of4}$	3.12	0.68	<.0001
$MDA_{Dassau\text{-}2of3}$ / $MDA_{Faccioli}$	1.51	0.33	0.6281
$MDA_{Dassau\text{-}2of3}$ / MDA_{Harvey}	1.11	0.25	1
$MDA_{Dassau\text{-}2of3}$ / $MDA_{Kölle\text{-}Ra}$	0.74	0.17	0.9325
$MDA_{Dassau\text{-}2of3}$ / MDA_{Popp}	0.50	0.12	0.1166
$MDA_{Dassau\text{-}2of3}$ / MDA_{Samadi}	0.26	0.07	0.0001
$MDA_{Dassau\text{-}2of3}$ / $MDA_{Turksoy}$	0.76	0.18	0.9616
$MDA_{Dassau\text{-}3of4}$ / $MDA_{Faccioli}$	0.49	0.10	0.0177
$MDA_{Dassau\text{-}3of4}$ / MDA_{Harvey}	0.36	0.08	0.0001
$MDA_{Dassau\text{-}3of4}$ / $MDA_{Kölle\text{-}Ra}$	0.24	0.05	<.0001
$MDA_{Dassau\text{-}3of4}$ / MDA_{Popp}	0.16	0.05	<.0001
$MDA_{Dassau\text{-}3of4}$ / MDA_{Samadi}	0.08	0.02	<.0001
$MDA_{Dassau\text{-}3of4}$ / $MDA_{Turksoy}$	0.24	0.05	<.0001
$MDA_{Faccioli}$ / MDA_{Harvey}	0.73	0.16	0.887
$MDA_{Faccioli}$ / $MDA_{Kölle\text{-}Ra}$	0.49	0.11	0.0436
$MDA_{Faccioli}$ / MDA_{Popp}	0.33	0.08	0.0002
$MDA_{Faccioli}$ / MDA_{Samadi}	0.17	0.05	<.0001
$MDA_{Faccioli}$ / $MDA_{Turksoy}$	0.50	0.11	0.0616
MDA_{Harvey} / $MDA_{Kölle\text{-}Ra}$	0.67	0.15	0.7167
MDA_{Harvey} / MDA_{Popp}	0.45	0.11	0.0336
MDA_{Harvey} / MDA_{Samadi}	0.24	0.07	<.0001
MDA_{Harvey} / $MDA_{Turksoy}$	0.69	0.16	0.7898
$MDA_{Kölle\text{-}Ra}$ / MDA_{Popp}	0.68	0.17	0.843
$MDA_{Kölle\text{-}Ra}$ / MDA_{Samadi}	0.35	0.10	0.0093
$MDA_{Kölle\text{-}Ra}$ / $MDA_{Turksoy}$	1.03	0.25	1
MDA_{Popp} / MDA_{Samadi}	0.52	0.16	0.4177
MDA_{Popp} / $MDA_{Turksoy}$	1.52	0.38	0.7785
MDA_{Samadi} / $MDA_{Turksoy}$	2.91	0.83	0.0062

FP/day

Contrast	IRR	SE	P value
$MDA_{Kölle\text{-}CGM}$ / $MDA_{Dassau\text{-}2of3}$	0.65	0.16	0.7044
$MDA_{Kölle\text{-}CGM}$ / $MDA_{Dassau\text{-}3of4}$	3.11	1.19	0.0748
$MDA_{Kölle\text{-}CGM}$ / $MDA_{Faccioli}$	0.28	0.06	<.0001
$MDA_{Kölle\text{-}CGM}$ / MDA_{Harvey}	1.47	0.44	0.9303
$MDA_{Kölle\text{-}CGM}$ / $MDA_{Kölle\text{-}Ra}$	1.17	0.32	0.9998
$MDA_{Kölle\text{-}CGM}$ / MDA_{Popp}	0.30	0.07	<.0001

$\text{MDA}_{\text{Kölle-CGM}}$ / $\text{MDA}_{\text{Samadi}}$	0.16	0.03	<.0001
$\text{MDA}_{\text{Kölle-CGM}}$ / $\text{MDA}_{\text{Turksoy}}$	1.75	0.55	0.6919
$\text{MDA}_{\text{Dassau-2of3}}$ / $\text{MDA}_{\text{Dassau-3of4}}$	4.78	1.75	0.0007
$\text{MDA}_{\text{Dassau-2of3}}$ / $\text{MDA}_{\text{Faccioli}}$	0.43	0.08	0.0001
$\text{MDA}_{\text{Dassau-2of3}}$ / $\text{MDA}_{\text{Harvey}}$	2.26	0.62	0.0742
$\text{MDA}_{\text{Dassau-2of3}}$ / $\text{MDA}_{\text{Kölle-}Ra}$	1.79	0.46	0.3489
$\text{MDA}_{\text{Dassau-2of3}}$ / MDA_{Popp}	0.47	0.09	0.0013
$\text{MDA}_{\text{Dassau-2of3}}$ / $\text{MDA}_{\text{Samadi}}$	0.25	0.04	<.0001
$\text{MDA}_{\text{Dassau-2of3}}$ / $\text{MDA}_{\text{Turksoy}}$	2.69	0.79	0.021
$\text{MDA}_{\text{Dassau-3of4}}$ / $\text{MDA}_{\text{Faccioli}}$	0.09	0.03	<.0001
$\text{MDA}_{\text{Dassau-3of4}}$ / $\text{MDA}_{\text{Harvey}}$	0.47	0.19	0.6508
$\text{MDA}_{\text{Dassau-3of4}}$ / $\text{MDA}_{\text{Kölle-}Ra}$	0.38	0.15	0.2272
$\text{MDA}_{\text{Dassau-3of4}}$ / MDA_{Popp}	0.10	0.03	<.0001
$\text{MDA}_{\text{Dassau-3of4}}$ / $\text{MDA}_{\text{Samadi}}$	0.05	0.02	<.0001
$\text{MDA}_{\text{Dassau-3of4}}$ / $\text{MDA}_{\text{Turksoy}}$	0.56	0.23	0.9053
$\text{MDA}_{\text{Faccioli}}$ / $\text{MDA}_{\text{Harvey}}$	5.26	1.32	<.0001
$\text{MDA}_{\text{Faccioli}}$ / $\text{MDA}_{\text{Kölle-}Ra}$	4.17	0.94	<.0001
$\text{MDA}_{\text{Faccioli}}$ / MDA_{Popp}	1.09	0.16	0.9997
$\text{MDA}_{\text{Faccioli}}$ / $\text{MDA}_{\text{Samadi}}$	0.57	0.07	0.0003
$\text{MDA}_{\text{Faccioli}}$ / $\text{MDA}_{\text{Turksoy}}$	6.25	1.68	<.0001
$\text{MDA}_{\text{Harvey}}$ / $\text{MDA}_{\text{Kölle-}Ra}$	0.79	0.24	0.9978
$\text{MDA}_{\text{Harvey}}$ / MDA_{Popp}	0.21	0.05	<.0001
$\text{MDA}_{\text{Harvey}}$ / $\text{MDA}_{\text{Samadi}}$	0.11	0.03	<.0001
$\text{MDA}_{\text{Harvey}}$ / $\text{MDA}_{\text{Turksoy}}$	1.19	0.40	0.9999
$\text{MDA}_{\text{Kölle-}Ra}$ / MDA_{Popp}	0.26	0.06	<.0001
$\text{MDA}_{\text{Kölle-}Ra}$ / $\text{MDA}_{\text{Samadi}}$	0.14	0.03	<.0001
$\text{MDA}_{\text{Kölle-}Ra}$ / $\text{MDA}_{\text{Turksoy}}$	1.50	0.48	0.9436
MDA_{Popp} / $\text{MDA}_{\text{Samadi}}$	0.53	0.07	<.0001
MDA_{Popp} / $\text{MDA}_{\text{Turksoy}}$	5.75	1.56	<.0001
$\text{MDA}_{\text{Samadi}}$ / $\text{MDA}_{\text{Turksoy}}$	10.86	2.84	<.0001

Δt

Contrast	Estimate	SE	P value
$\text{MDA}_{\text{Kölle-CGM}}$ / $\text{MDA}_{\text{Dassau-2of3}}$	0.32	0.14	0.2976
$\text{MDA}_{\text{Kölle-CGM}}$ / $\text{MDA}_{\text{Dassau-3of4}}$	0.34	0.14	0.2907
$\text{MDA}_{\text{Kölle-CGM}}$ / $\text{MDA}_{\text{Faccioli}}$	0.23	0.14	0.7582
$\text{MDA}_{\text{Kölle-CGM}}$ / $\text{MDA}_{\text{Harvey}}$	0.31	0.14	0.348
$\text{MDA}_{\text{Kölle-CGM}}$ / $\text{MDA}_{\text{Kölle-}Ra}$	0.12	0.14	0.997
$\text{MDA}_{\text{Kölle-CGM}}$ / MDA_{Popp}	−0.77	0.14	<.0001
$\text{MDA}_{\text{Kölle-CGM}}$ / $\text{MDA}_{\text{Samadi}}$	−0.77	0.14	<.0001
$\text{MDA}_{\text{Kölle-CGM}}$ / $\text{MDA}_{\text{Turksoy}}$	0.19	0.14	0.8991
$\text{MDA}_{\text{Dassau-2of3}}$ / $\text{MDA}_{\text{Dassau-3of4}}$	0.01	0.14	1
$\text{MDA}_{\text{Dassau-2of3}}$ / $\text{MDA}_{\text{Faccioli}}$	−0.09	0.14	0.9991
$\text{MDA}_{\text{Dassau-2of3}}$ / $\text{MDA}_{\text{Harvey}}$	−0.01	0.14	1
$\text{MDA}_{\text{Dassau-2of3}}$ / $\text{MDA}_{\text{Kölle-}Ra}$	−0.22	0.14	0.8097
$\text{MDA}_{\text{Dassau-2of3}}$ / MDA_{Popp}	−1.10	0.14	<.0001
$\text{MDA}_{\text{Dassau-2of3}}$ / $\text{MDA}_{\text{Samadi}}$	−1.09	0.14	<.0001
$\text{MDA}_{\text{Dassau-2of3}}$ / $\text{MDA}_{\text{Turksoy}}$	−0.14	0.14	0.9864

$MDA_{Dassau\text{-}3of4}$ / $MDA_{Faccioli}$	−0.11	0.14	0.9981
$MDA_{Dassau\text{-}3of4}$ / MDA_{Harvey}	−0.03	0.14	1
$MDA_{Dassau\text{-}3of4}$ / $MDA_{Kölle\text{-}Ra}$	−0.23	0.14	0.7878
$MDA_{Dassau\text{-}3of4}$ / MDA_{Popp}	−1.11	0.14	<.0001
$MDA_{Dassau\text{-}3of4}$ / MDA_{Samadi}	−1.10	0.14	<.0001
$MDA_{Dassau\text{-}3of4}$ / $MDA_{Turksoy}$	−0.15	0.14	0.9803
$MDA_{Faccioli}$ / MDA_{Harvey}	0.08	0.14	0.9997
$MDA_{Faccioli}$ / $MDA_{Kölle\text{-}Ra}$	−0.12	0.14	0.993
$MDA_{Faccioli}$ / MDA_{Popp}	−1.00	0.14	<.0001
$MDA_{Faccioli}$ / MDA_{Samadi}	−0.99	0.14	<.0001
$MDA_{Faccioli}$ / $MDA_{Turksoy}$	−0.04	0.14	1
MDA_{Harvey} / $MDA_{Kölle\text{-}Ra}$	−0.21	0.14	0.8531
MDA_{Harvey} / MDA_{Popp}	−1.08	0.14	<.0001
MDA_{Harvey} / MDA_{Samadi}	−1.08	0.14	<.0001
MDA_{Harvey} / $MDA_{Turksoy}$	−0.12	0.14	0.9925
$MDA_{Kölle\text{-}Ra}$ / MDA_{Popp}	−0.88	0.14	<.0001
$MDA_{Kölle\text{-}Ra}$ / MDA_{Samadi}	−0.87	0.14	<.0001
$MDA_{Kölle\text{-}Ra}$ / $MDA_{Turksoy}$	0.08	0.14	0.9996
MDA_{Popp} / MDA_{Samadi}	0.01	0.14	1
MDA_{Popp} / $MDA_{Turksoy}$	0.96	0.14	<.0001
MDA_{Samadi} / $MDA_{Turksoy}$	0.96	0.14	<.0001

Pairwise contrasts are shown on the natural effect scale of each model: odds ratios (sensitivity model), incidence rate ratios (false-positive model), and estimated differences (detection-time model). SE denotes standard error, and p-values are Tukey-adjusted.

Abbreviations: IRR, incidence rate ratios; SE, standard error; MDA, meal detection algorithm; FP/day, false positives per day; Δt, detection time.

REFERENCES

1. Brummer, J., Glasbrenner, C., Hechenbichler Figueroa, S., Koehler, K. & Höchsmann, C. Continuous glucose monitoring for automatic real-time assessment of eating events and nutrition: a scoping review. *Front Nutr* **10**, 1308348 (2023).
2. Popp, C. J. *et al.* Objective Determination of Eating Occasion Timing: Combining Self-Report, Wrist Motion, and Continuous Glucose Monitoring to Detect Eating Occasions in Adults With Prediabetes and Obesity. *J Diabetes Sci Technol* **18**, 266–272 (2024).
3. Kölle, K., Biester, T., Christiansen, S., Fougner, A. L. & Stavdahl, O. Pattern Recognition Reveals Characteristic Postprandial Glucose Changes: Non-Individualized Meal Detection in Diabetes Mellitus Type 1. *IEEE J Biomed Health Inform* **24**, 594–602 (2020).
4. Dassau, E., Bequette, B. W., Buckingham, B. A. & Doyle, F. J. Detection of a meal using continuous glucose monitoring: implications for an artificial beta-cell. *Diabetes Care* **31**, 295–300 (2008).
5. Ornetzeder, C. *et al.* Feasibility of Fully Closed Loop Insulin Delivery in Type 2 Diabetes. in *2019 IEEE Conference on Control Technology and Applications (CCTA)* 906–913 (2019). doi:10.1109/CCTA.2019.8920591.
6. Palisaitis, E., El Fathi, A., von Oettingen, J. E., Haidar, A. & Legault, L. A Meal Detection Algorithm for the Artificial Pancreas: A Randomized Controlled Clinical Trial in Adolescents With Type 1 Diabetes. *Diabetes Care* **44**, 604–606 (2021).
7. de Carvalho, D., Kaymak, U., Van Gorp, P. & van Riel, N. Data-driven meal events detection using blood glucose response patterns. *BMC Med Inform Decis Mak* **23**, 282 (2023).
8. Fushimi, E., Colmegna, P., De Battista, H., Garelli, F. & Sánchez-Peña, R. Unannounced meal analysis of the ARG algorithm. in *2019 American Control Conference (ACC)* 4740–4745 (2019). doi:10.23919/ACC.2019.8814719.
9. Atlas, E., Nimri, R., Miller, S., Grunberg, E. A. & Phillip, M. MD-logic artificial pancreas system: a pilot study in adults with type 1 diabetes. *Diabetes Care* **33**, 1072–1076 (2010).

10. Fathi, A. E., Palisaitis, E., Boulet, B., Legault, L. & Haidar, A. An Unannounced Meal Detection Module for Artificial Pancreas Control Systems. in *2019 American Control Conference (ACC)* 4130–4135 (2019). doi:10.23919/ACC.2019.8814932.
11. Godoy, J. L., Sereno, J. E. & Rivadeneira, P. S. Meal detection and carbohydrate estimation based on a feedback scheme with application to the artificial pancreas. *Biomedical Signal Processing and Control* **68**, 102715 (2021).
12. Mosquera-Lopez, C. *et al.* Enabling fully automated insulin delivery through meal detection and size estimation using Artificial Intelligence. *npj Digit. Med.* **6**, 1–7 (2023).
13. Ibrahim, M., Beneyto, A., Contreras, I. & Vehi, J. An ensemble machine learning approach for the detection of unannounced meals to enhance postprandial glucose control. *Comput Biol Med* **171**, 108154 (2024).
14. Ramkissoon, C. M., Herrero, P., Bondia, J. & Vehi, J. Unannounced Meals in the Artificial Pancreas: Detection Using Continuous Glucose Monitoring. *Sensors (Basel)* **18**, 884 (2018).
15. Dovc, K. *et al.* Faster Compared With Standard Insulin Aspart During Day-and-Night Fully Closed-Loop Insulin Therapy in Type 1 Diabetes: A Double-Blind Randomized Crossover Trial. *Diabetes Care* **43**, 29–36 (2020).
16. Bertrand, L., Cleyet-Marrel, N. & Liang, Z. Recognizing Eating Activities in Free-Living Environment Using Consumer Wearable Devices. *Engineering Proceedings* **6**, 58 (2021).
17. Palacios, V., Woodbridge, D. M.-K. & Fry, J. L. Machine Learning-based Meal Detection Using Continuous Glucose Monitoring on Healthy Participants: An Objective Measure of Participant Compliance to Protocol. *Annu Int Conf IEEE Eng Med Biol Soc* **2021**, 7032–7035 (2021).
18. Bertrand, L., Cleyet-Marrel, N. & Liang, Z. The Role of Continuous Glucose Monitoring in Automatic Detection of Eating Activities. in *2021 IEEE 3rd Global Conference on Life Sciences and Technologies (LifeTech)* 313–314 (2021). doi:10.1109/LifeTech52111.2021.9391849.

19. Presseller, E. K., Parker, M. N., Zhang, F., Manasse, S. & Juarascio, A. S. Continuous glucose monitoring as an objective measure of meal consumption in individuals with binge-spectrum eating disorders: A proof-of-concept study. *Eur Eat Disord Rev* **32**, 828–837 (2024).
20. Pellizzari, E. *et al.* Automatic identification of unreported meals from continuous glucose monitoring data in individuals after bariatric surgery using a template matching algorithm. *Sci Rep* **15**, 7797 (2025).
21. Hoyos, J. D. *et al.* Characterization of glycemic patterns in type 1 diabetes without insulin or meal input data. in *2022 10th International Conference on Systems and Control (ICSC)* 576–581 (2022). doi:10.1109/ICSC57768.2022.9993837.
22. Faccioli, S. *et al.* Super-twisting-based meal detector for type 1 diabetes management: Improvement and assessment in a real-life scenario. *Comput Methods Programs Biomed* **219**, 106736 (2022).
23. Harvey, R. A., Dassau, E., Zisser, H., Seborg, D. E. & DoyleIII, F. J. Design of the Glucose Rate Increase Detector: A Meal Detection Module for the Health Monitoring System. *J Diabetes Sci Technol* **8**, 307–320 (2014).
24. Bergman, R. N., Phillips, L. S. & Cobelli, C. Physiologic evaluation of factors controlling glucose tolerance in man: measurement of insulin sensitivity and beta-cell glucose sensitivity from the response to intravenous glucose. *J Clin Invest* **68**, 1456–1467 (1981).
25. Dalla Man, C., Rizza, R. A. & Cobelli, C. Meal Simulation Model of the Glucose-Insulin System. *IEEE Transactions on Biomedical Engineering* **54**, 1740–1749 (2007).
26. Samadi, S. *et al.* Automatic Detection and Estimation of Unannounced Meals for Multivariable Artificial Pancreas System. *Diabetes Technol Ther* **20**, 235–246 (2018).
27. Turksoy, K. *et al.* Meal Detection in Patients With Type 1 Diabetes: A New Module for the Multivariable Adaptive Artificial Pancreas Control System. *IEEE J Biomed Health Inform* **20**, 47–54 (2016).
